# Clinical Characteristics and Prognostic Risk Factors of Patients With Proven Invasive Pulmonary Aspergillosis: A Single-Institution Retrospective Study

**DOI:** 10.3389/fmed.2021.756237

**Published:** 2021-12-23

**Authors:** Xiang Tong, Tao Liu, Kexin Jiang, Dongguang Wang, Sitong Liu, Ye Wang, Hong Fan

**Affiliations:** ^1^Department of Respiratory Medicine and Critical Care Medicine, West China Hospital/West China School of Medicine, Sichuan University, Chengdu, China; ^2^Clinical Medicine of Eight-Year Program, West China Hospital/West China School of Medicine, Sichuan University, Chengdu, China

**Keywords:** clinical characteristics, imaging features, prognosis, risk factor, IPA

## Abstract

**Background:** The mortality and burden of medical costs associated with invasive pulmonary aspergillosis (IPA) is very high. Currently, the clinical features and prognostic factors of patients with proven IPA are not very clear, especially in the Chinese population. In this retrospective analysis, we aimed to identify the clinical features and prognostic factors of patients with proven IPA.

**Methods:** The diagnostic criteria for proven IPA were based on the international consensus of the EORTC/MSG. Data of patients with proven IPA at the West China Hospital of Sichuan University between January 2012 and December 2018 were collected. The optimal cut-off value of continuous variables was determined by Receiver Operating Characteristic curve and maximum Youden's index. Finally, using the Cox regression analysis to identify correlations between the clinical parameters associated with morbidity.

**Results:** A total of 117 patients with proven IPA were included in the study, and 32 (27.4%) patients died during the follow-up period. Compared with the survivor group, elderly, patients with comorbidities, and patients undergoing chemotherapy and the level of inflammatory biomarkers [erythrocyte sedimentation rate, platelet count, interleukin-6, C-reactive protein (CRP)] in the non-survivor group were higher, while the albumin level was lower (*P* = 0.018). The imaging features were consolidation, nodules, cavities, pleural effusion, ground-glass shadows, and halo signs in order. Overall, 41.0% patients had mixed imaging features. The results suggested the most appropriate cut-off value of age and CRP were 60 years and 14.1 mg/L, respectively. The multivariate Cox regression analysis suggested that advanced age (>60 years) [hazard ratio (HR): 10.7, confidence interval (CI): 2.5–44.9, *P* < 0.001), undergoing chemotherapy (HR: 9.5, CI: 2.7–32.9, *P* < 0.001), presence of pleural effusion (HR: 5.74, CI: 1.6–20.8, *P* = 0.008), and increased CRP levels (>14.1 mg/L) (HR: 6.3, CI: 1.2–34.3, *P* = 0.033) were risk factors for all-cause mortality in patients with proven aspergillosis.

**Conclusions:** This study showed that the prognosis of proven IPA is poor, and the age >60 years, undergoing chemotherapy, pleural effusion on CT image, and CRP levels >14.1 mg/L may be as risk factors for mortality in patients with proven IPA. large samples and real-world studies are needed to confirm these results in the future.

## Introduction

*Aspergillus spp*. is ubiquitous in the environment. The global burden of pulmonary aspergillosis cannot be underestimated. Invasive aspergillosis is the least common, with 0.2 million cases each year. However, it likely represents only 50–60% of actual cases (1). Aspergillus mainly affects immunocompromised individuals, has an extremely high mortality rate between 40 and 90% (2). In addition, Zilberberg et al. showed that the hospital cost of patients with aspergillosis as the principal diagnosis increased from $440 million in 2004 to $590 million in 2013 after adjusting for inflation (3). Despite the increased awareness, increasing number of susceptible individuals and subspecies, and improvement in antemortem diagnosis, China has reported a steady increase in the number of aspergillosis cases for decades (4). In addition, the unaffordability of antifungal treatment, ineffectiveness of antifungal treatment, or failure of patients to adhere to antifungal treatment has resulted in severe outcomes, which have had a huge impact on public health. Nonetheless, epidemiological data on aspergillosis remain scarce in China.

Early diagnosis of pulmonary aspergillosis remains a challenge and should be based on the integration of clinical, microbiological, and radiological data (5). In fact, there is a large proportion of patients with “probable diagnoses” because there are no pathological results. Few studies have focused on the clinical features and prognostic factors of patients with proven aspergillosis. Therefore, in this study, we retrospectively investigated the case data of patients with invasive pulmonary aspergillosis (IPA) confirmed by pathology (proven IPA) at the West China Hospital of Sichuan University to gain a better understanding of its clinical features and risk factors. In addition, this study also provides a reference basis for establishing a prospective multicenter cohort of pulmonary aspergillosis in Western China.

## Materials and Methods

### Patients, Setting and Definitions

This study retrospectively collected the available medical records and data of patients with proven IPA at the West China Hospital, Sichuan University, from January 2012 to December 2018. The diagnostic criteria for IPA were mainly based on the international consensus of the European Organization of Treatment and Research of Cancer and the Mycosis Study Group (EORTC/MSG) (6). Proven IPA identification requires histopathological or cytopathological examination of a specimen obtained by needle aspiration or biopsy in which hyphae or melanized yeast-like forms are seen accompanied by evidence of associated tissue damage, or positive culture for *Aspergillus* from a sample obtained by sterile procedure from the lung (6).

In this study, all patients underwent lung or bronchoscopy biopsy (including needle aspiration), and the pathological findings (*Aspergillus* infection) were confirmed by two experienced pathologists. All included patients were inpatients at the West China Hospital of Sichuan University, while outpatients and patients who underwent pathological consultations from other hospitals were excluded. Follow-up and prognostic data were obtained *via* telephone interviews or hospital electronic information systems. If a patient's follow-up data were not available or a patient could not be contacted by telephone after more than three attempts, the patient was excluded. All-cause death was defined as the endpoint of this study. The final follow-up of this study was on January 1, 2020.

This retrospective study was approved by the Biomedical Ethics Committee of the West China Hospital of Sichuan University (No. 2021-305). We confirm that all the experiment protocol for involving human data was in accordance to guidelines of national/international/institutional and Declaration of Helsinki in the manuscript. The Biomedical Ethics Committee waived the requirement for informed consent (No. 2021-305), and all original data used in the analyses were anonymized and de-identified.

### Data Collection

The history information system (HIS) was queried to identify all patients with proven IPA. Demographic information, clinical parameters, underlying diseases, and medications for aspergillosis were included in the data extracted from the HIS. Laboratory examinations were obtained from the initial results of patients within 3 days after admission, including white blood cell count (WBC), albumin (ALB) count, erythrocyte sedimentation rate (ESR), platelet count, C-reactive protein (CRP) level, glycated hemoglobin, interleukin (IL)-6, galactomannan (GM) test, and pathogen culture. Radiological features were identified in chest computed tomography (CT) images by two experienced radiologists. All included patients were followed-up to obtain the follow-up data.

### Statistical Analysis

Continuous variables with normal distribution are expressed as mean ± standard deviation, and the *t*-test was used for comparisons between two groups. Continuous variables with non-normal distribution are described as median with interquartile (25th percentile, 75th percentile), and the Mann-Whitney U-test was used for comparisons among groups. Frequency and percentage were used to describe categorical variables, which were analyzed using the chi-square test or Fisher's exact test. The optimal cut-off value of continuous variables and their sensitivity and specificity were determined by Receiver Operating Characteristic (ROC) curve and maximum Youden's index. Statistical significance was set at *p* < 0.05, and all statistical analyses were two-sided. Kaplan–Meier survival curves and Cox regression analysis were used to identify the risk factors associated with all-cause death in patients with aspergillosis, and significance was determined using the log-rank test. Statistical analysis was performed using the SPSS software (version 21.0; IBM Corp., Armonk, NY, USA).

## Results

### Study Population

**Figure 1:**
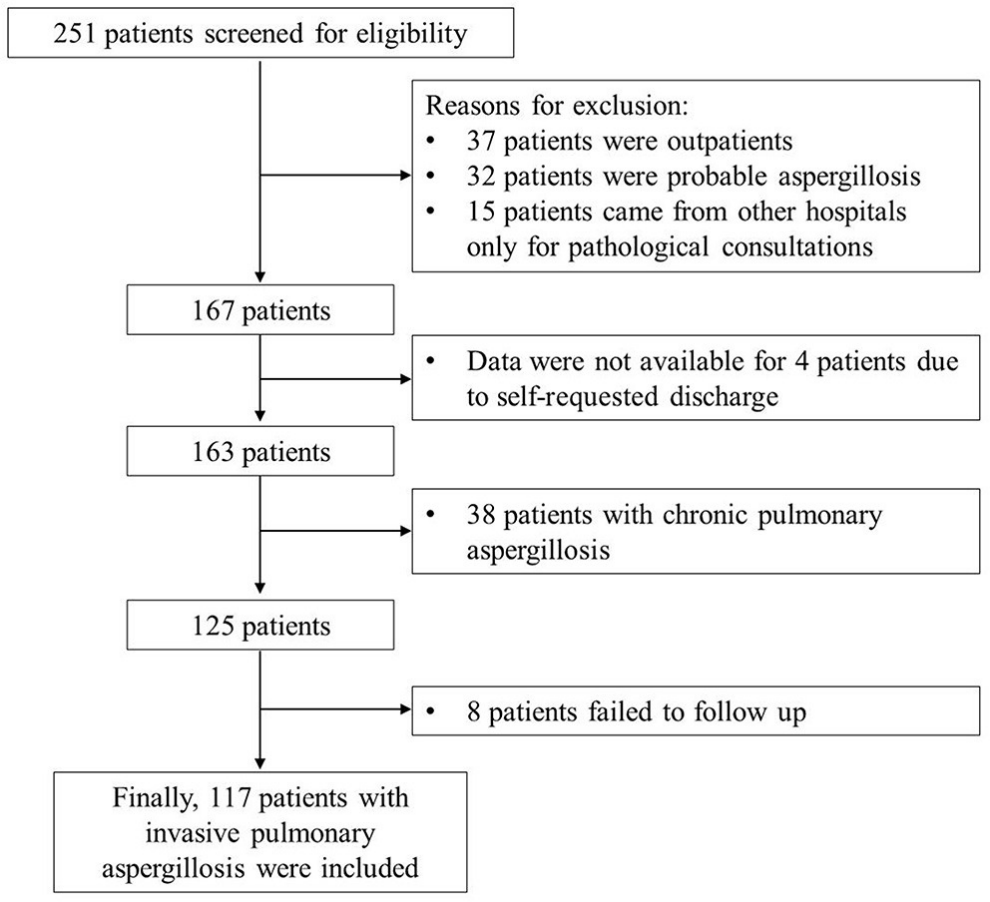
Flow diagram for the selection of patients.

All 125 patients with proven IPA who met the inclusion criteria (6) were enrolled, and eight patients were lost to follow-up. Finally, 117 patients were included in this retrospective analysis (Figure 1). Among them, 74 (63.2%) patients were males, the average age was 52.6 ± 12.9 years. There were 85 (72.6%) survivors and 32 (27.4%) non-survivors. The most common underlying comorbidities were diabetes mellitus (25.6%), malignancy (20.5%), bronchiectasis (17.1%), and chronic obstructive pulmonary disease (COPD) (12.0%). It is worth noting that one patient was HIV positive, one patient underwent lung organ transplantation, and 39 (27.4%) patients had two or more comorbidities. Additionally, 16 patients (13.7%) had a history of tuberculosis, and 15 patients (12.8%) underwent chemotherapy. In terms of etiology, a total of 31 patients (26.5%) were positive for *Aspergillosis spp*. of sputum or bronchoalveolar lavage fluid (BALF) culture, of which two patients were combined with mucor.

### Difference in the Clinical Characteristics Between the Survival and Non-survival Groups

The clinical characteristics of the survival and non-survival (overall) groups are summarized in Table 1. Patients with proven aspergillosis who died were more likely to be older (*P* < 0.001). Symptoms such as dyspnea (*P* = 0.007), comorbidities such as COPD (*P* = 0.002), and malignancy (*P* < 0.001) were more common in the non-survival group. There were more all-cause deaths were associated with two or more comorbidities (*P* = 0.020). Moreover, patients who did not undergo chemotherapy had a higher chance of survival (*P* < 0.001). In terms of laboratory test results, patients with lower WBC (*P* = 0.004), higher ALB (*P* = 0.018), lower ESR (*P* = 0.007), lower platelet (PLT) (*P* = 0.037), lower IL-6 (*P* = 0.018), and lower CRP (*P* = 0.003) were likely to survive.

**Table 1:** Baseline and clinical characteristics of all patients with proven IPA.

**Parameters**	**Survivors** **(*n* = 85)**	**Non-survivors** **(*n* = 32)**	***P*-value**
Male/Female, *n*	49/36	25/7	0.053
Age, years (mean ± SD)	49.1 ± 12.2	61.8 ± 10.1	<0.001
Chemotherapy, *n* (%)	2 (2.4)	13 (40.6)	<0.001
History of tuberculosis, *n* (%)	13 (15.3)	3 (9.4)	0.552
Symptoms, *n* (%)			
Cough	64 (75.3)	30 (93.8)	0.035
Fever	30 (35.3)	9 (28.1)	0.516
Hemoptysis	46 (54.1)	16 (50.0)	0.681
Dyspnea	10 (11.8)	11 (34.4)	0.007
Chest congestion	15 (17.6)	8 (25.0)	0.438
Others	15 (17.6)	2 (6.3)	0.225
Comorbidity, *n* (%)			
COPD	4 (4.7)	10 (31.3)	<0.001
Diabetes mellitus	21 (24.7)	9 (28.1)	0.813
Bronchiectasis	18 (21.2)	2 (6.3)	0.060
Malignancy	7 (8.2)	17 (53.1)	<0.001
Two or more diseases	18 (21.2)	14 (43.8)	0.020
WBC (median (interquartile), × 10^9^ cells/L)	6.4 (4.6–9.6)	8.2 (6.8–11.8)	0.004
ALB (mean ± SD, g/L)	36.8 ± 7.7	33.1 ± 6.4	0.018
ESR (mean ± SD, mm/h)	50.9 ± 32.1	73.9 ± 37.1	0.007
PLT (mean ± SD, × 10^9^ cells/L)	208.6 ± 102.8	254.8 ± 106.4	0.037
CRP (median (interquartile), mg/L)	14.1 (4.0–78.7)	86.3 (18.6–157.5)	0.003
IL-6 (median (interquartile), pg/ml)	17.5 (6.8–43.0)	113.4 (10.4–196.2)	0.018
Glycated hemoglobin (mean ± SD)	8.3 ± 2.5	8.0 ± 2.2	0.785
GM test positive, *n* (%)	13 (15.3)	4 (12.5)	1.0
Imaging site, *n* (%)			
Left lobe	62 (72.9)	17 (53.1)	0.049
Right lobe	22 (25.9)	15 (46.9)	0.044
More than three lesions	34 (40.0)	15 (46.9)	0.534
Imaging features, *n* (%)			
Consolidation	45 (52.9)	26 (81.3)	0.006
Cavity	36 (42.4)	10 (31.3)	0.297
Halo sign	9 (10.6)	5 (15.6)	0.525
Nodule	42 (49.4)	17 (53.1)	0.837
Ground-glass opacity	15 (17.6)	5 (15.6)	1.0
Pleural effusion	16 (18.8)	15 (46.9)	0.004
Mixed	33 (38.8)	15 (46.9)	0.528
Antibiotic use, *n* (%)	29 (34.1)	16 (50.0)	0.138
Etiology positive, *n* (%)	22 (25.9)	9 (28.1)	0.817

*IPA, invasive pulmonary aspergillosis; SD, standard deviation; COPD, chronic obstructive pulmonary disease; WBC, white blood cell; ALB, albumin; ESR, erythrocyte sedimentation rate; PLT, platelet; CRP, C-reactive protein; IL-6, interleukin-6; GM, galactomannan*.

### Difference in the Radiologic Characteristics Between the Survival and Non-survival Groups

The most common abnormalities were consolidation (*n* = 71, 60.7%), nodules (*n* = 59, 50.4%), cavity (*n* = 46, 39.3%), pleural effusion (*n* = 31, 26.5%), ground-glass opacity (*n* = 20, 17.1%), and the halo sign (*n* = 14, 12.0%). Overall, 48 (41.0%) patients had mixed imaging features. For all-cause death analysis, on comparison of the different imaging features, survivors were less likely to present with consolidation (*P* = 0.006) and pleural effusion (*P* = 0.004). The differences in other imaging features between the groups are shown in Table 1.

### Difference in the Treatment Drugs Between the Survival and Non-survival Groups

The treatment strategy is mainly treatment with voriconazole alone (*n* = 94, 80.3%), followed by amphotericin B (*n* = 11, 9.4%), Echinocandins (*n* = 6, 5.1%), Posaconazole (*n* = 4, 3.4%), and Itraconazole (*n* = 2, 1.7%). The number of patients treated with two drugs was small (*n* = 2, 1.7%). As shown in Table 2, for overall cause death analysis, there was no significant difference between the survival and non-survival groups, regardless of whether they were treated with a single-agent or combination therapy.

**Table 2:** Difference in the treatment drugs between the survival and non-survival groups.

**Drugs** **(*n*, %)**	**Survivors** **(*n* = 85)**	**Non-survivors** **(*n* = 32)**	***P*-value**
Voriconazole	70 (82.4)	24 (75.0)	0.278
Amphotericin B	8 (9.4)	3 (9.4)	1.0
Echinocandins	3 (3.5)	3 (9.4)	0.343
Posaconazole	3 (3.5)	1 (3.1)	1.0
Itraconazole	1 (1.1)	1 (3.1)	0.481
Combination therapy	2 (2.4)	0 (0)	0.560

### Determination of Optimal Cut-Off Value of Continuous Variables

In this study, the ROC analyses and maximum Youden's index were used to determine the most appropriate cut-off value of continuous variables for overall survival in patients with proven IPA. The results suggested the most appropriate cut-off value of age, WBC, ALB, ESR, PLT, IL-6, and CRP were 60 years, 6.6 × 10^9^ cells/L, 38.8 g/L, 86.0 mm/h, 190.0 × 10^9^ cells/L, 44.6 pg/ml, 14.1 mg/L, respectively. According to the cut-off value, we converted these continuous variables into dichotomous variables, and used them in the subsequent Cox regression analysis.

### Correlations Between All-Cause Death and Parameters of Patients With Proven IPA

**Figure 2:**
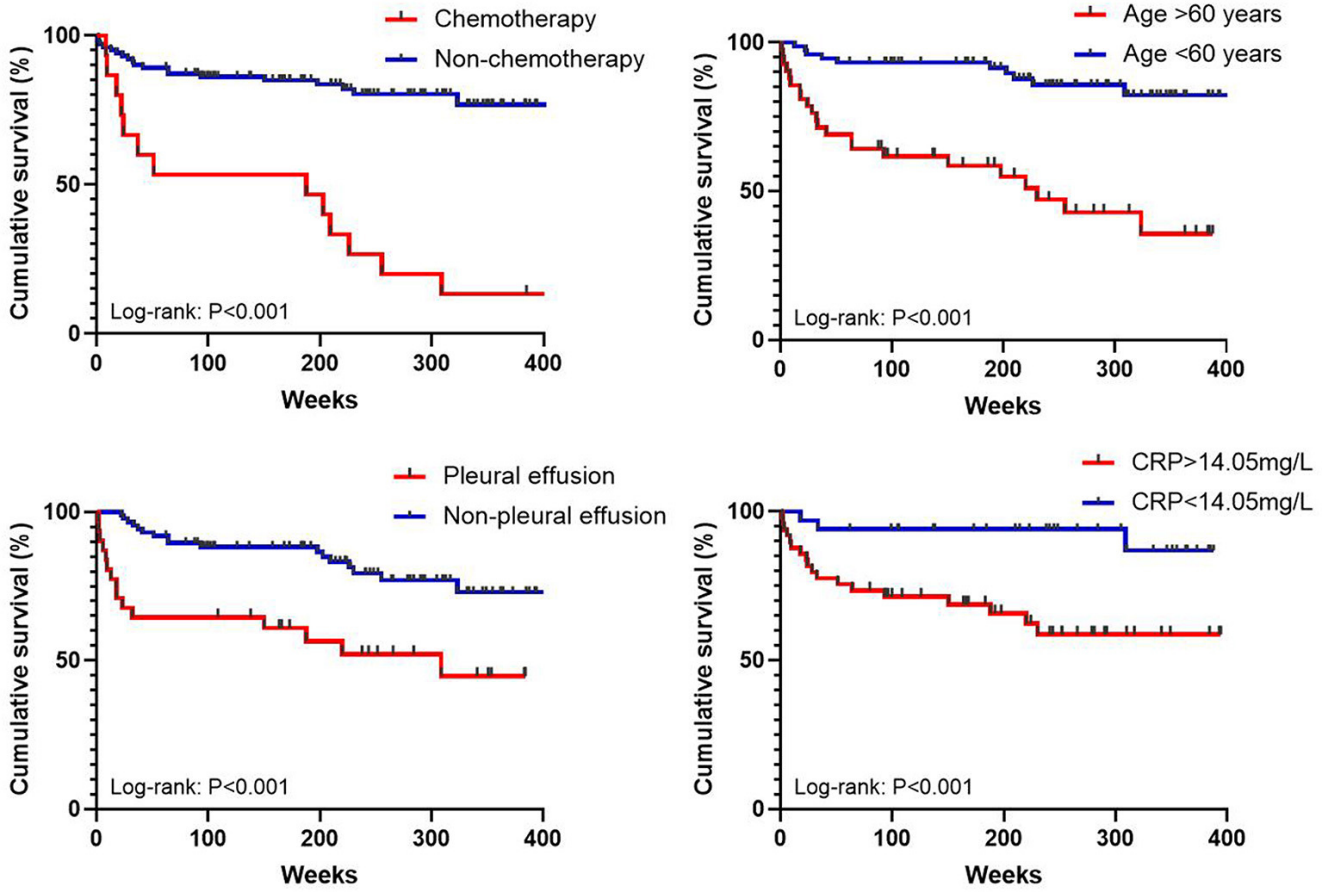
Kaplan–Meier curves of overall patient survival according to age category, chemotherapy status, presence of pleural effusion, and CRP levels.

The median follow-up time was 1,559 days, and the all-cause mortality over the follow-up period was 27.4% in patients with proven IPA. For all-cause death analysis, in the multivariate Cox regression analysis (Table 3), when the cut-off value of age was set at 60 years and the CRP count cut-off was 14.1 mg/L, age [hazard ratio (HR): 10.7, confidence interval (CI): 2.5–44.9, *P* < 0.001), undergoing chemotherapy (HR: 9.5, CI: 2.7–32.9, *P* < 0.001), pleural effusion on CT image (HR: 5.74, CI: 1.6–20.8, *P* = 0.008), and CRP count (HR: 6.3, CI: 1.2–34.3, *P* = 0.033) were found to be independent risk factors for mortality. Additionally, it cannot be proven that consolidation on the CT image, albumin level, ESR level, or having two or more comorbidities is correlated with survival. Kaplan–Meier curves of overall patient survival according to age category, chemotherapy status, presence of pleural effusion, and CRP levels are shown in Figure 2.

**Table 3:** Multivariate cox regression analysis of all-cause death over the entire follow-up period in patients with proven IPA.

**Parameters**	**HR (95% CI)**	***P*-value**
Age (>60 years)	10.67 (2.54–44.90)	<0.001
Chemotherapy	9.46 (2.72–32.94)	<0.001
Consolidation	1.23 (0.15–9.81)	0.846
Pleural effusion	5.74 (1.59–20.78)	0.008
Albumin (<38.75)	1.03 (0.18–5.93)	0.973
CRP (>14.05)	6.31 (1.16–34.33)	0.033
ESR (>86.0)	1.19 (0.34–4.16)	0.785
Two or more comorbidity	0.91(0.22–3.77)	0.897

*IPA, invasive pulmonary aspergillosis; CRP, C-reactive protein; ESR, erythrocyte sedimentation rate; HR, hazard ratio; CI, confidence interval*.

## Discussion

In this single-center study, 117 patients with proven IPA were included. The main research results are as follows: first, 27.4% patients died during the follow-up period, and comorbidities were more common among the patients who died. The all-cause mortality of patients with proven IPA in this study is similar to that reported in previous studies involving Chinese patients (7, 8); second, chest CT showed that pulmonary consolidation and pleural effusion were more common in the non-survival group; third, advanced age (>60 years), increased CRP levels (>14.1 mg/L), undergoing chemotherapy, and presence of pleural effusion were risk factors for all-cause mortality in patients with proven IPA.

In this study, the most common underlying comorbidities were diabetes mellitus, malignancy, bronchiectasis, and COPD. Uncontrolled diabetes patients are usually considered to be immunocompromised because hyperglycemia may lead to impaired neutrophil function, antioxidant system, and humoral immune function (9). Several previous studies have shown that the incidence and mortality of pulmonary aspergillosis are significantly increased in immunocompromised patients (10, 11). Aspergillus can colonize and grow in the injured area of the lung in patients with bronchiectasis, and fungal infection can also lead to bronchiectasis. Aspergillosis is common not only in severely immunosuppressed hosts, but also in patients with structural lung diseases, including bronchiectasis and COPD (12, 13). In addition, studies have shown that diabetes and bronchiectasis are risk factors for the prognosis of aspergillosis (14, 15).

We found that the levels of inflammatory biomarkers (WBC, CRP, IL-6, and ESR) were significantly increased in the non-survivor group, and the multivariate Cox regression analysis found that the elevated CRP level (>14.1 mg/L) was closely correlated with poor prognosis of patients with proven aspergillosis (HR: 6.3, CI: 1.2–34.3, *P* = 0.033), which was similar to the results of a previous study (16). Chai et al. found that the continuously elevated levels of IL-6, IL-8, and CRP may be an early predictor of the adverse outcome of invasive aspergillosis, which can be used for the early identification of patients with poor response to antifungal therapy, and may help in the optimization of antimicrobial therapy (16). Pang et al. found that the CRP level was closely correlated with the prognosis of aspergillosis after the secondary analysis of two multicenter randomized cohorts. However, the study found that an increase in PLT may be positively correlated with the survival of patients with aspergillosis (17). However, our study found that although the number of PLT in the non-survival group was higher than that in the survival group at admission, they were all within the normal reference range.

Advanced age and chemotherapy are correlated with the impairment of immune function. Therefore, they are recognized risk factors for infectious diseases, including aspergillosis (7, 11, 18–20). In this study, we also found that advanced age and chemotherapy increased the overall mortality of patients with proven IPA (HR: 10.7, CI: 2.5–44.9, *P* < 0.001; HR: 9.5, CI: 2.7–32.9, *P* < 0.001; respectively). It is worth noting that 24 patients with malignant tumors were included in this study; however, only two patients with hematological malignancies were included. It is well known that hematologic malignancies and stem cell transplantation are important risk factors for IPA (21). The main reason for the inclusion of a small number of patients with hematological malignancies in our study may be that many of these patients cannot undergo puncture or biopsy, and usually rely on clinical data and etiological examination results; therefore, these severe patients were likely diagnosed with hematological malignancies and excluded from this study. In addition, in this study, it was found that the positive rate of pathogenic culture in patients with proven IPA was relatively low (26.5%). In combination with all the above findings, we recommend that when patients have a high mortality risk and can tolerate biopsy, pathological diagnosis should be made as early as possible to identify the disease, so as to avoid underestimating the disease and leading to increased mortality.

In this study, we found that there was no difference in the distribution of lung lesions between the two groups; however, consolidation and pleural effusion were more common in the non-survival group. Interestingly, our results suggest that pleural effusion could increase the overall mortality of patients with proven IPA, which is consistent with previous reports (22, 23). Nivoix et al. suggested that the proportion of invasive aspergillosis patients with pleural effusion was as high as 47.8%, while the proportion of aspergillosis patients with pleural effusion in our study was 26.5%. However, another study reported that the proportion of patients with invasive aspergillosis and pleural effusion was only 2.2% (24). Although we were unable to confirm whether Aspergillus was present in the pleural effusion, our results and those of previous studies suggest that pleural effusion increased the overall mortality of patients with IPA, which may be due to the more severe inflammatory reaction and disease condition in IPA patients with pleural effusion.

This study has several limitations. First, the GM assay is broadly accepted as a valuable tool for the diagnosis of patients with IPA (25, 26), including patients in the ICU (27). Additionally, the GM samples from the BALF were more than GM samples from the serum (28). However, the BALF-GM test was officially launched in our hospital in 2016; therefore, some patients did not have the BALF-GM test results. There may be bias in the results value of the serum and BALF-GM tests in patients with proven IPA. Second, as the nature limited value of retrospective studies on long-term prognosis, it is difficult to make further hierarchical analysis on some possible influencing factors, which may have a certain impact on the real results. Third, it is difficult to further analyze the cause of aspergillosis-specific death in detail; therefore, all-cause mortality was used in this study.

## Conclusions

In summary, our study suggests that advanced age (>60 years) (HR: 10.7, *P* < 0.001), undergoing chemotherapy (HR: 9.5, *P* < 0.001), presence of pleural effusion (HR: 5.74, *P* = 0.008), and elevated CRP levels (>14.1 mg/L) (HR: 6.3, *P* = 0.033) may be risk factors for all-cause mortality in patients with proven IPA. We strongly suggested that pathological diagnosis should be made as early as possible for patients with a high mortality risk and who can tolerate to biopsy. In the future, large samples and real-world studies are needed to verify these results.

## Data Availability Statement

The original contributions presented in the study are included in the article/supplementary material, further inquiries can be directed to the corresponding authors.

## Ethics Statement

The studies involving human participants were reviewed and approved by Biomedical Ethics Committee of the West China Hospital of Sichuan University. The patients/participants provided their written informed consent to participate in this study.

## Author Contributions

HF, XT, and YW designed the study, coordinated the study, and directed its implementation. XT, TL, KJ, DW, and SL collected data and conducted the follow-up work. XT, TL, and KJ wrote the manuscript. All the authors read and approved the final manuscript. All authors contributed to the article and approved the submitted version.

## Funding

This project was funded by China Postdoctoral Science Foundation (2020M673259) and Post-Doctor Research Project, West China Hospital, Sichuan University (2020HXBH013).

## Conflict of Interest

The authors declare that the research was conducted in the absence of any commercial or financial relationships that could be construed as a potential conflict of interest.

## Publisher's Note

All claims expressed in this article are solely those of the authors and do not necessarily represent those of their affiliated organizations, or those of the publisher, the editors and the reviewers. Any product that may be evaluated in this article, or claim that may be made by its manufacturer, is not guaranteed or endorsed by the publisher.

## Abbreviations

IPA: invasive pulmonary aspergillosis
CRP: C-reactive protein
CPA: chronic pulmonary aspergillosis
HIS: history information system
ALB: albumin
ESR: erythrocyte sedimentation rate
IL-6: interleukin-6
PLT: platelet
GM: galactomannan
CT: computed tomography
ROC: receiver operator characteristic curve
COPD: chronic obstructive pulmonary disease
BALF: bronchoalveolar lavage fluid
HR: hazard ratio
CI: confidence interval.

## References

[1] Bongomin F Gago S Oladele RO Denning DW. Global and multi-national prevalence of fungal diseases-estimate precision. J Fungi. (2017) 3. 10.3390/jof3040057 29371573 PMC5753159

[2] Lin SJ Schranz J Teutsch SM. Aspergillosis case-fatality rate: systematic review of the literature. Clin Infect Dis. (2001) 32:358–66. 10.1086/318483 11170942

[3] Zilberberg MD Harrington R Spalding JR Shorr AF. Burden of hospitalizations over time with invasive aspergillosis in the United States, 2004-2013. BMC Public Health. (2019) 19:591. 10.1186/s12889-019-6932-9 31101036 PMC6525423

[4] Chen M Xu Y Hong N Yang Y Lei W Du L. Epidemiology of fungal infections in China. Front Med. (2018) 12:58–75. 10.1007/s11684-017-0601-0 29380297

[5] Ullmann AJ Aguado JM Arikan-Akdagli S Denning DW Groll AH Lagrou K. Diagnosis and management of Aspergillus diseases: executive summary of the 2017 ESCMID-ECMM-ERS guideline. Clin Microbiol Infect. (2018) 24 Suppl 1:e1–e38. 10.1016/j.cmi.2018.01.002 29544767

[6] Donnelly JP Chen SC Kauffman CA Steinbach WJ Baddley JW Verweij PE. Revision and update of the consensus definitions of invasive fungal disease from the european organization for research and treatment of cancer and the mycoses study group education and research consortium. Clin Infect Dis. (2020) 71:1367–76. 10.1093/cid/ciz1008 31802125 PMC7486838

[7] Lao M Zhang K Zhang M Wang Q Li J Su L. Clinical features and co-infections in invasive pulmonary aspergillosis in elderly patients. Infect Drug Resist. (2020) 13:3525–34. 10.2147/IDR.S273946 33116671 PMC7567571

[8] Sun KS Tsai CF Chen SC Huang WC. Clinical outcome and prognostic factors associated with invasive pulmonary aspergillosis: an 11-year follow-up report from Taiwan. PLoS ONE. (2017) 12:e0186422. 10.1371/journal.pone.0186422 29049319 PMC5648178

[9] Akash MSH Rehman K Fiayyaz F Sabir S Khurshid M. Diabetes-associated infections: development of antimicrobial resistance and possible treatment strategies. Arch Microbiol. (2020) 202:953–65. 10.1007/s00203-020-01818-x 32016521 PMC7223138

[10] Dunbar A Schauwvlieghe A Rijnders BJA. Influenza and invasive aspergillosis in immunocompromised patients. Clin Infect Dis. (2019) 69:2037. 10.1093/cid/ciz316 30982862

[11] Maschmeyer G Haas A Cornely OA. Invasive aspergillosis: epidemiology, diagnosis and management in immunocompromised patients. Drugs. (2007) 67:1567–601. 10.2165/00003495-200767110-00004 17661528

[12] De Soyza A Aliberti S. Bronchiectasis and aspergillus: how are they linked? Med Mycol. (2017) 55:69–81. 10.1093/mmy/myw109 27794529

[13] Hammond EE McDonald CS Vestbo J Denning DW. The global impact of aspergillus infection on COPD. BMC Pulm Med. (2020) 20:241. 10.1186/s12890-020-01259-8 32912168 PMC7488557

[14] Zhang L Che C. Clinical manifestations and outcome analysis of invasive pulmonary aspergillosis infection: a retrospective study in 43 nonneutropenic patients. J Int Med Res. (2019) 47:5680–8. 10.1177/0300060519874901 31566035 PMC6862873

[15] Iqbal N Irfan M Zubairi AB Jabeen K Awan S Khan JA. Clinical manifestations and outcomes of pulmonary aspergillosis: experience from Pakistan. BMJ Open Respir Res. (2016) 3:e000155. 10.1136/bmjresp-2016-000155 28074136 PMC5174800

[16] Chai L Netea MG Teerenstra S Earnest A Vonk AG Schlamm HT. Early proinflammatory cytokines and C-reactive protein trends as predictors of outcome in invasive Aspergillosis. J Infect Dis. (2010) 202:1454–62. 10.1086/656527 20879853

[17] Pang L Zhao X Dickens BL Lim JT Cook AR Netea MG. Using routine blood parameters to anticipate clinical outcomes in invasive aspergillosis. Clin Microbiol Infect. (2020) 26:781.e1–e8. 10.1016/j.cmi.2019.10.019 31669427

[18] Ledoux MP Guffroy B Nivoix Y Simand C Herbrecht R. Invasive pulmonary aspergillosis. Semin Respir Crit Care Med. (2020) 41:80–98. 10.1055/s-0039-3401990 32000286

[19] Castle SC Uyemura K Fulop T Makinodan T. Host resistance and immune responses in advanced age. Clin Geriatr Med. (2007) 23:463–79. 10.1016/j.cger.2007.03.005 17631228 PMC7135540

[20] Bow EJ. Invasive aspergillosis in cancer patients. Oncology. (2001) 15:1035–9. 11548975

[21] Nucci M Anaissie E. Fungal infections in hematopoietic stem cell transplantation and solid-organ transplantation–focus on aspergillosis. Clin Chest Med. (2009) 30:295–306. 10.1016/j.ccm.2009.03.001 19375636

[22] Cordonnier C Ribaud P Herbrecht R Milpied N Valteau-Couanet D Morgan C. Prognostic factors for death due to invasive aspergillosis after hematopoietic stem cell transplantation: a 1-year retrospective study of consecutive patients at French transplantation centers. Clin Infect Dis. (2006) 42:955–63. 10.1086/500934 16511759

[23] Nivoix Y Velten M Letscher-Bru V Moghaddam A Natarajan-Amé S Fohrer C. Factors associated with overall and attributable mortality in invasive aspergillosis. Clin Infect Dis. (2008) 47:1176–84. 10.1086/592255 18808352

[24] Koehler P Salmanton-García J Gräfe SK Koehler FC Mellinghoff SC Seidel D. Baseline predictors influencing the prognosis of invasive aspergillosis in adults. Mycoses. (2019) 62:651–8. 10.1111/myc.12926 31066092

[25] Yu Y Zhu C Shen H Liu C Guo R Gao Y. Galactomannan detection in bronchoalveolar lavage fluid corrected by urea dilution for the diagnosis of invasive pulmonary aspergillosis among nonneutropenic patients. J Thorac Dis. (2019) 11:465–76. 10.21037/jtd.2019.01.07 30962990 PMC6409274

[26] Savio J Menon NR Sudharma AR Jairaj V Mathew J. Galactomannan assay and invasive pulmonary aspergillosis-comparison of the test performance at an in-house and the kit cut-off. J Clin Diagn Res. (2016) 10:Dc01–4. 10.7860/JCDR/2016/19175.8310 27656435 PMC5028428

[27] Schroeder M Simon M Katchanov J Wijaya C Rohde H Christner M. Does galactomannan testing increase diagnostic accuracy for IPA in the ICU? A prospective observational study. Crit Care. (2016) 20:139. 10.1186/s13054-016-1326-1 27160692 PMC4862077

[28] Zhou W Li H Zhang Y Huang M He Q Li P. Diagnostic value of galactomannan antigen test in serum and bronchoalveolar lavage fluid samples from patients with nonneutropenic invasive pulmonary aspergillosis. J Clin Microbiol. (2017) 55:2153–61. 10.1128/JCM.00345-17 28446576 PMC5483917

